# Supplementary material for: Identification and functional analysis of non-coding regulatory small RNA FenSr3 in Bacillus amyloliquefaciens LPB-18
Source: PeerJ. 2023 May 15;11:e15236. doi: 10.7717/peerj.15236 (PMC10194069; doi:10.7717/peerj.15236)
Supplement: Supplemental Information 4 [file peerj-11-15236-s004.zip › KO/CK-vs-T1_map/map04137.html]

KEGG PATHWAY: Mitophagy - animal - Reference pathway


|  |  |
| --- | --- |
| **Mitophagy - animal - Reference pathway** |  |

[
Pathway menu
| Organism menu
| Pathway entry
| Show description
| User data mapping
]

Mitochondria act as the energy powerhouse of the cell, and are essential for eukaryotic cells to grow and function normally. However, deleterious byproducts of oxidative phosphorylation process called reactive oxidative species (ROS) lead to mitochondrial dysfunction. If the damage is too excessive to be repaired, such mitochondria are selectively recognized and targeted for degradation by a specific mode of autophagy, termed mitophagy. The loss of the mitochondrial membrane potential can induce mitophagy, involving the kinase PINK1 and the E3 ligase Parkin. PINK1 serves as the sensor for the mitochondrial depolarization and recruits Parkin, followed by ubiquitin-dependent recruitment of mitophagy receptors. There are also several PINK1/Parkin-independent mitophagy pathways, in which a group of LIR-containing receptors are required in response to different stimuli. Mitophagy contributes to the maintenance of a healthy mitochondrial network and the prevention of programmed cell death.

|  |  |  |
| --- | --- | --- |
| Reference pathway | 184% 150% 122% 100% 82% 67% 55% | 图片下载 |
